# Measuring patient-reported outcomes in advanced gastric cancer

**DOI:** 10.3332/ecancer.2013.351

**Published:** 2013-09-16

**Authors:** Jianming Xu, TR Jeffry Evans, Cheryl Coon, Kati Copley-Merriman, Yun Su

**Affiliations:** 1 Beijing 307 Hospital Cancer Center, Beijing 100071, China; 2 University of Glasgow, Beatson Institute for Cancer Research, Garscube Estate, Switchback Road, Bearsden, Glasgow G61 1BD, UK; 3 RTI Health Solutions, 200 Park Offices Drive, Research Triangle Park, NC 27709, USA; 4 Current address: Adelphi Values, 290 Congress Street, Boston, MA 02210, USA; 5 RTI Health Solutions, 3005 Boardwalk Street, Suite 105, Ann Arbor, MI 48108, USA; 6 Bristol-Myers Squibb, 5 Research Parkway, Wallingford, CT 06492, USA

**Keywords:** gastric cancer, patient outcome assessment, outcome measures, digestive signs, symptoms

## Abstract

**Background:**

Gastric cancer (GC), one of the most common cancers in the world, is often diagnosed at an advanced stage and associated with a poor prognosis. Quality of life and patient-reported outcomes (PROs) are important considerations when treating GC patients. The aim of this study was to identify existing PRO instruments that would be appropriate for use in GC trials.

**Methods:**

Data were obtained from a systematic literature review and interviews with clinical experts. A literature search was conducted using OVID (EMBASE and MEDLINE) and yielded 1,008 abstracts; 92 assessed PROs in an advanced GC.

**Results:**

Key symptoms and functional impacts identified through the literature and expert input included abdominal pain or pain at the site of distant metastases, dysphagia and other symptoms related to eating, and digestive symptoms. The liver and lungs were the most frequent locations of metastases, leading to dyspnea, abdominal fullness, and jaundice. Symptoms related to changes in bowel habits appeared to be more frequent and pronounced in Asian patients, possibly due to the higher prevalence of GC in the body of the stomach in this population. The five most commonly used PRO instruments were identified, but their validity in advanced-stage GC patients remains unclear.

**Conclusions:**

The symptoms and functional impacts identified here should be confirmed with robust input from advanced-stage GC patients. Optimal measurement of PROs in GC should account for patient burden and possible differences between Asian and non-Asian patients.

## Introduction

Gastric cancer (GC) and oesophagogastric junction (OGJ) adenocarcinoma are among the leading causes of cancer mortality in the world (736,000 deaths in 2008), accounting for approximately 24,000 and 10,400 newly diagnosed cancers each year in the United States and the United Kingdom, respectively [1]. Although there has been a decline in the incidence of GC in Western countries over the past few decades, this decline has been accompanied by an increase in the incidence of tumours of the OGJ and a shift toward poorly differentiated adenocarcinomas [2].

There is a significant geographical variation in the incidence of GC, with China having the highest incidence rate worldwide [3], resulting in 352,000 deaths in 2008 [4]. More than half of all the cases in the world occur in Western Pacific countries, including Australia, China, Japan, Korea, and Vietnam. The early stages of GC are often asymptomatic. The most common symptoms at diagnosis include abdominal pain, dysphagia, weight loss, nausea, and vomiting [5, 6], and the symptoms of advanced GC are often indistinguishable from benign disorders. Thus, the diagnosis is frequently made when the cancer is at an advanced stage, which is associated with a poor prognosis [7]. The estimated five-year survival rate is just 7% in patients with stage-IV disease [8].

The vast majority (80–90%) of patients present with locally advanced or metastatic disease that is unsuitable for curative resection. Combination chemotherapy results in a significant survival advantage in patients with advanced GC when compared with the best supportive care in randomised clinical trials [9–11]. There is currently no chemotherapy regimen that is universally accepted as a standard of care worldwide. The most frequently used regimens are either doublets of platinum with a fluoropyrimidine or a triplet drug regimen combining either epirubicin or docetaxel with a platinum and a fluoropyrimidine [12–14]. Nevertheless, the overall survival (OS) remains disappointing in these patients, with a median of 10–11 months [14], although the addition of trastuzumab to a chemotherapy backbone has further improved the OS to beyond one year in the subset of patients with HER2-positive tumours [15]. Consequently, quality of life (QOL) remains an important consideration when planning treatment in patients with an advanced disease.

Assessment of QOL, symptoms, and other related patient-reported outcomes (PROs) is increasingly performed in oncology clinical trials. In patients with advanced disease, the goals of the treatment are to achieve palliative benefits and improvement in survival. Therefore, it is crucial to understand the impact of treatment on symptoms, functioning, and QOL. Understanding the benefit–risk profile associated with oncology treatments is also of increasing importance to regulatory agencies, and PRO assessment is an important source of information regarding the positive and negative impacts of treatment from the patient’s perspective. Within a clinical trial setting, data on symptoms and symptom burden through PRO instruments can supplement the understanding of treatment benefits provided by the efficacy outcomes such as OS, progression-free survival (PFS), and response rate. PRO data may provide evidence of improvement in QOL or functional status related to reduced symptoms, even in the absence of improvement in the efficacy outcomes.

A survey of oncologists found that 51% of the respondents reported that PRO study findings had influenced their recommendations for treatment [16]. From the patient’s perspective, they are becoming more involved in decision making for their own treatment and subsequent treatment compliance. This trend is particularly relevant as an increasing number of oncology therapies are administered orally at home, and noncompliance with treatment may be a significant cause of treatment failure [17]. Finally, baseline PRO instruments have been shown to predict survival in many cancer types, including advanced GC [18]. Consequently, the aim of this study was to identify the existing PRO instruments that would be appropriate to apply in clinical trials in patients with advanced or metastatic GC.

## Methods

A structured literature search with predefined inclusion criteria was conducted using OVID, which accesses EMBASE, OVID MEDLINE, and EconLit. The search strategy combined disease terms for advanced metastatic stage-III or -IV GC or stomach cancer, with terms describing the outcomes of interest, including QOL, patient perspective, preference, patient satisfaction, activities of daily living, burden of illness, symptom, PROs, well-being, pain, functional status, and health status. The search was conducted in 2011 with no limits on publication date, study type, or language; 1,008 articles were identified. Abstracts of all 1,008 articles were reviewed, and those articles deemed relevant were retrieved for full text review. From the 1,008 abstracts reviewed, 92 relevant studies met the inclusion criteria and were included in this review. Studies were considered relevant to this review if they described the development, validation, or use of a PRO instrument in a chemotherapy clinical study or an observational study in advanced GC. PRO instruments described in these studies included assessments of QOL, functional status, health status, treatment satisfaction, or GC symptoms.

Additional potentially relevant studies were identified from the reference lists of the retrieved articles. Information on the association of GC symptoms and tumour location was informed by the clinical experience of the authors. Further information about PRO instruments used to assess outcomes in GC was obtained from the summaries of GC treatments as described in the *Physician’s Desk Reference*, the European Medicines Agency’s summary of product characteristics, and clinicaltrials.gov.

## Results

### Key symptoms and functional impacts

Key symptoms and functional impacts identified through the literature and expert input included abdominal pain or pain from metastatic disease sites, dysphagia and other eating-related symptoms, and digestive symptoms. The metastases in different sites and extents usually lead to different symptoms, such as dyspnea, abdominal fullness, and occasionally, jaundice, etc. Symptoms related to the change in bowel habit appeared to be more frequent and pronounced in Asian patients, possibly due to a higher prevalence of GC in the body of the stomach in this population.

### Association of patient-reported symptoms, location of GC tumour, and geographic location

Regional differences in the location of GC tumours have been noted in many studies and in clinical practice. Proximal GC is more common in Western countries and is associated with poorer outcomes [19]. While the incidence of GC in general is declining around the world, this is generally limited to cancers of the cardia [20]. In contrast, distal tumours are more common in Eastern countries, and it has been speculated that this incidence rate is associated with food additives consumed more frequently in Asian cultures [20, 21]. These patterns also hold for ethnic groups within the United States, with cancers of the cardia being more common in whites and cancers of the antrum and pylorus being more common in African Americans and Asian and Pacific Islanders [22]. Geographic regions can be considered predictors of safety outcomes, such as neutropaenia and diarrhoea, which have been shown to have lower incidence rates in Asian trials versus non-Asian trials [23].

### PRO instruments available for use in advanced GC

Despite the use of the PRO instruments in advanced-staged GC clinical trials in the published literature, no product label in the *Physician’s Desk Reference *for a GC treatment referenced a PRO instrument at the time this review was conducted. A search of the European Medicines Agency’s summary of product characteristics identified four products, all docetaxel based, that are indicated for advanced-stage GC and that referenced a PRO instrument [specifically, the European Organisation for Research and Treatment QOL Questionnaire-Core 30 (EORTC QLQ-C30)]. Although these four products are indicated for advanced-stage GC, the EORTC QLQ-C30 was used only in docetaxel-based studies in metastatic breast cancer. Thus, the sensitivity of the instrument in measuring treatment differences for GC cannot be determined from these products’ labels.

A search of clinicaltrials.gov identified 97 studies in advanced-stage GC that used a PRO instrument, and 17 PRO instruments specific to GC were identified. Combined with the results of previously published literature, the PRO instruments reported to be used in a GC population were as follows:

(a) Health-related QOL: SF-36 Health Survey and the McGill QOL.
(b) General cancer: EORTC QLQ-C30.
(c) GC specific: European Organisation for Research and Treatment QOL Questionnaire-GC Module (EORTC QLQ-STO22).
(d) Other cancer specific: European Organisation for Research and Treatment QOL Questionnaire-Oesophageal, European Study for Pancreatic Cancer-QOL Questionnaire, the MD Anderson Symptom Inventory-Gastrointestinal (MDASI-GI), and Functional Assessment of Cancer Therapy (FACT)-Oesophageal.
(e) Cancer treatment: QOL-Anticancer Drugs.
(f) Symptoms: FACT-Fatigue, FACT-Anaemia, Brief Pain Inventory, and Edmonton Symptom Assessment Scale Score.
(g) Emotional functioning: Hospital Anxiety and Depression Scale, State–Trait Anxiety Inventory, Depression Status Inventory, Positive and Negative Affect Schedules, and Profile of Mood States.
(h) Other: Medical Coping Modes Questionnaire, Linear Analogue Self-Assessment, and Time Without Symptom and Toxicity.
(i) Utility: EuroQol-5 Dimension (EQ-5D) and Health Utilities Index 3.

### Sensitivity of PRO instruments identified in the literature

The EORTC QLQ-C30 has been used frequently to assess outcomes in a GC population. This instrument has been able to demonstrate treatment differences or improvements on physical, role, cognitive, emotional, and social functioning, as well as fatigue, nausea and vomiting, pain, dyspnea, diarrhoea, constipation, insomnia, appetite loss, numbness or paraesthesia, and global health status. Treatment differences or improvements have also been shown in QOL on the EQ-5D visual analogue scale (VAS) and in anxiety and depression as reported on the Hospital Anxiety and Depression Scale. In addition to their use in clinical trials, many of the PRO instruments listed here were included in the observational or cross-sectional studies to predict outcomes, rather than to distinguish between treatments or assess efficacy within the treatment.

In the published psychometric evaluations, the instruments registered differences between groups that were known to differ on clinical outcomes. The EORTC QLQ-STO22 was able to distinguish between clinically distinct groups (curative versus palliative) on dysphagia, eating restrictions, taste problems, and body image [24]. The FACT-Gastric Cancer (FACT-Ga) showed significant differences between groups identifying themselves as having improved, worsened, or remained the same over a three-month study [25]. The MADSI-GI has been shown to be sensitive enough to distinguish the functional status in GI cancer patients and to differentiate among GC groups defined by disease level [26]. Finally, in one study, all the scales and items from the EORTC QLQ-C30 were able to discriminate among GC survivors, GC patients undergoing curative treatment and those undergoing palliative treatment, and GC patients categorised by the performance status [27].

## Breadth of concepts measured

During the course of the review, five cancer-specific PRO instruments were identified as being potentially useful in assessing the treatment benefit in clinical trials of advanced GC: the EORTC QLQ-C30, the EORTC QLQ-STO22, the FACT-General (FACT-G), the FACT-Ga, and the MDASI-GI. These instruments were selected because of their demonstrated psychometric properties and evidence of appropriate patient involvement in the generation or confirmation of the questionnaire items—except the FACT-G, which was selected because of its intended use in combination with the FACT-Ga. Patient involvement in the development process supports an instrument’s face and content validity, in line with the recommendations of the US Food and Drug Administration’s guidance for PROs [28].

Three instruments were GC or GI cancer specific: the EORTC QLQ-STO22 [29], the FACT-Ga [30], and the MDASI-GI [26]. Two instruments were developed for cancer in general, to be used in conjunction with cancer-type-specific instruments: the EORTC QLQ-C30 [31] and the FACT-G [32].

The EORTC QLQ-STO22 and the FACT-Ga were specifically developed for patients with GC. The EORTC QLQ-STO22 was generated from a comprehensive literature search and physician interviews into a set of candidate concepts. Interviews conducted by the instrument developers with 24 health professionals and 58 GC patients (from a range of European countries, disease stages, and treatments) provided feedback on the scope of concepts represented, and cognitive debriefing with 115 additional GC patients tested the wording and appropriateness of the items. The FACT-Ga was developed from patient inputs (along with physician input) solicited for item generation, scale construction, content validation, and scale refinement [30]. While the patient characteristics for the initial patient input work of the FACT-Ga were not reported, later cognitive interviews in Canada and Japan included patients with GC and cancer of the GC cardia.

The EORTC QLQ-C30 and FACT-G, on the other hand, were developed for use in a broad range of cancer patients. The authors of the EORTC QLQ-C30, Blazeby *et al *[24], received positive feedback during their cognitive interviews with GC patients for the EORTC QLQSTO22. There is no evidence of content validity for the FACT-G in GC. Because the MDASI-GI is intended for GI cancer in general, it does not limit patient involvement to those with GC. However, the authors used a large group of patients with GI cancer to develop the instrument (item generation, item reduction, and cognitive debriefing), including 23 patients specifically with GC.

The two generic cancer instruments, EORTC QLQ-C30 and FACT-G, assess PROs related to well-being and physical, emotional, and social functioning. The GC-specific instruments, EORTC QLQ-STO22 and FACT-Ga, assess GC symptoms of pain, dysphagia, problems with eating, and reflux or upper GI symptoms. The MDASI-GI assesses GI symptoms, general cancer symptoms, and interference.

In addition to measuring aspects of health-related QOL, the five PRO instruments measure a number of GC symptoms. Table 1provides a list of symptoms addressed in these instruments, listed as they would be used in practice (core plus tumour-specific module, where appropriate). None of the instruments captures the full range of GC symptoms, but the inclusion of the GC-specific modules (EORTC QLQSTO22 and FACT-Ga) and the more general GI-specific instrument (MDASI-GI) allow for a wide portion of symptoms to be covered. GC symptoms not present in any of the identified PRO instruments included haematemesis or melaena, changes in bowel habits, cough or haemoptysis, fever or sweats, pruritus, jaundice, need to eat frequent small meals, and bone pain.

None of the identified PRO instruments comprehensively cover all of the QOL and symptom concepts that could be relevant to patients with advanced GC. The most comprehensive coverage of GC symptoms and their potential impact is provided by the EORTC QLQ-C30 supplemented with the EORTC QLQ-STO22; however, these two instruments combined have 52 items, which is likely to be burdensome for patients with advanced or metastatic GC. Perhaps because of the number of items in the pair of instruments, the randomised clinical trials in advanced GC that we identified used only the EORTC QLQ-C30 for assessing all the cancer types. This approach suggested that these trials did not assess concepts that may be important to patients with GC. Given these limitations and the content of the MDASI-GI, the 24-item MDASI-GI is a reasonable option that covers general cancer symptoms, relevant GI symptoms, and interference; also, the MDASI-GI can be completed by most of the patients in 2–3 min.

In terms of using these instruments in patients with advanced-stage GC, those instruments developed for use in GC studied patients with a range of disease stages, give breadth of coverage of the concepts. Thus, while the items may not have been targeted to advanced-stage GC patients specifically, the instruments should be appropriate for measuring a wide variety of disease states, which is appropriate for assessing change over time in this population.

## Psychometric measurement properties

Table 2provides an overview of the five instruments selected for the possible use in GC studies, on the basis of each instrument’s characteristics and the psychometric evidence it identifies. In some of the instruments, it should be noted that the psychometric properties were evaluated in patients with GC but the samples were not limited to only those with advanced-stage GC.

Table 2shows that four of the five PRO instruments for use in GC demonstrated some levels of psychometric validity in a GC population, while the psychometric properties of the FACT-G have yet to be assessed (the instrument was included to complement the GC module, the FACT-Ga). Both internal consistency reliability and test–retest reliability have been demonstrated by the EORTC QLQ-STO22 and FACT-Ga in GC, while only internal consistency reliability has been demonstrated by the MDASI-GI and EORTC QLQ-C30 in GC. Each of the four instruments has documented validity (construct, discriminant, concurrent, or criterion), and the EORTC QLQ-STO22, the EORTC QLQ-C30, and the FACT-Ga have assessed ability to detect change in a GC population. In the studies that reported response rates, missing data did not appear to be a problem for the EORTC QLQ-C30, the EORTC QLQ-STO22, or the MDASI-GI.

### Appropriateness in light of regulatory guidance

In general, the five instruments considered for assessing the symptoms and impacts of GC demonstrated acceptable psychometric measurement properties. Some evidence of reliability and validity was available for each instrument, although the stand-alone FACT-G has not been formally evaluated in advanced GC.

The developers of these instruments did not document any information regarding data saturation, which is often required by regulatory agencies for justifying the content (both included and excluded) of items on an instrument. Additionally, while the developers designed their qualitative studies to include a sampling of countries, disease stages, and treatments, patients from a limited number of countries were included for all but the FACT-Ga, which conducted qualitative work in both North America and Asia. Prior to a regulatory submission, collaboration with the developers of the chosen instrument would be required to properly document both qualitative and quantitative evidence of the appropriateness of the instrument in an advanced GC population, with cognitive interviews in this population likely needed as a formal evidence of concept saturation.

Regulatory agencies, and particularly the US Food and Drug Administration, have expressed a preference for a 24-h recall period when posing PRO questions. The concern is that the response to an item that requires a longer period may involve calculations that differ across patients (e.g., how do patients average their experience over the past week?). In light of this, the MDASI-GI has an advantage of having a 24-h recall period, while the other four instruments refer to the past week or past seven days.

### Value of PRO assessment in advanced GC clinical trials

A French phase-2 randomised controlled trial of 5-fluorouracil and leucovorin (LV5FU2) in combination with irinotecan versus LV5FU2 mono-therapy or LV5FU2-cisplatin in 134 patients with metastatic GC adenocarcinoma found that after six months of treatment, patients in all the three groups experienced significant increases in the EORTC QLQ-C30 domains role, emotional, social, pain, insomnia, appetite loss, and global health. Furthermore, with the threshold of a ten-point or greater change in EORTC QLQ-C30 domain score as clinically significant, LV5FU2-irinotecan significantly improved on the role functioning, social functioning, and appetite loss domains; LV5FU2 monotherapy improved on the emotional functioning, nausea/vomiting, pain, insomnia, appetite loss, and constipation domains; and LV5FU2-cisplatin improved on the global health, physical functioning, role functioning, emotional functioning, fatigue, pain, insomnia, and appetite loss domains but worsened on dyspnea. LV5FU2 monotherapy was associated with poorer clinical outcomes and fewer QOL benefits. While the two LV5FU2 combination therapies improved a number of EORTC QLQ-C30 domains, LV5FU2-irinotecan was found to be superior to LV5FU2-cisplatin for PFS, and LV5FU2-cisplatin worsened dyspnea, which was not observed in LV5FU2-irinotecan [38].

Additionally, a large phase-3 randomised controlled trial comparing docetaxel and cisplatin plus fluorouracil (DCF) to cisplatin and fluorouracil (CF) in 445 patients with metastatic or locally recurrent GC or OGJ adenocarcinoma in 16 countries found that clinical outcomes favouring DCF were supported by PRO assessments. PROs, as assessed by the EORTC QLQ-C30 and the EQ-5D, showed that the time to global health deterioration was significantly longer for DCF. DCF also preserved EQ-5D VAS scores and EORTC QLQ-C30 scores related to physical functioning, social functioning, nausea/vomiting, pain, and appetite loss for a longer time versus CF, supporting the clinical superiority of DCF [39].

In a phase-3 randomised controlled trial of 333 European patients with metastatic or locally recurrent adenocarcinoma of the stomach or OGJ, the two treatments, irinotecan, folinic acid, and 5-fluorouracil (5-FU) (IF); and cisplatin and 5-FU (CF), were not differentiated by significant clinical outcomes (e.g., time to progression); however, scores on the EORTC QLQ-C30 and EQ-5D supported the superiority of IF with respect to PROs [40]. Between-group differences were significant for the EORTC QLQ-C30 and EQ-5D. Follow-up scores were significantly better in the IF arm as compared with the CF arm for EORTC QLQ C30 physical functioning (*P *= 0.003) and nausea/vomiting (*P*= 0.026) and for EQ-5D health state (*P*= 0.018) and VAS (*P*= 0.002).

Park *et al *[41] also reported the results of a clinical trial finding significant differences in treatment based on PRO assessments but not clinical endpoints. A single-centre phase-2 randomised controlled trial in Korea of paclitaxel plus 5-FU (PF) versus docetaxel plus 5-FU (DF) as first-line therapy for metastatic GC found that failure-free survival, OS, and ORR did not significantly differ between the two treatment arms. The EORTC QLQ-C30 found that both the arms showed clinically significant (ten-point change or greater) improvement in role, emotional, and constipation domains. Additionally, the PF arm was associated with clinically significant improvement in pain, dyspnea, and diarrhoea, and the DF arm with clinically significant improvement in cognitive functioning but clinically significant worsening in appetite.

### Additional considerations for using PROs in advanced GC

When selecting an instrument for use in a population with advanced-stage cancer, the number of items in the instrument and its frequency of administration should be balanced to minimise patient burden and missing data. For example, completion rates for the EORTC QLQ-C30 in metastatic GC patients decreased over the course of treatment but were generally lower for patients who discontinued treatment and who experienced tumour progression [38]. In instruments developed for a more general population, administration is often as a daily diary, which may satisfy regulatory requirements of a short recall period. However, this type and frequency of administration are likely too burdensome for GC patients. Patients with the poorest QOL might be less likely to complete the PRO, which would lead to bias in the missing data. Furthermore, if PRO responses are solicited too frequently at home, the power provided by the additional data to be modelled longitudinally will likely be negated by the amount of missing patient responses. To balance the need for complete data as often as possible, one approach would be to administer PRO instruments at each study visit, prior to treatment. Feasibility studies also could be conducted to document the amount of effort required for completion.

One advantage held by the FACT-Ga is that it was developed concurrently in North America (Canada) and Asia (Japan). Because of the prevalence of GC in Asian populations, it is important to identify an appropriate instrument in terms of both the relevance of concepts across cultures and the suitability of translations. Good research practices in the linguistic translation and cultural adaptation of PRO instruments recommend forward and backward translation, reconciliation, harmonisation, patient cognitive interviews, and psychometric equivalency testing to ensure that the concepts measured by the instrument are relevant across the cultures and translations result in scores that mean the same thing regardless of language version [42]. Invariably, the PRO instruments used in Asian studies are linguistically translated from PRO instruments developed for implementation in Western countries. However, language translations might not be sufficient for cultural adaptation or for ensuring that the instrument’s concepts are important and clearly defined for an Asian population. With distal GC being more common in Asian populations and associated with symptoms of vomiting, heartburn, and indigestion, the EORTC QLQ-STO22 may be the most appropriate PRO instrument in this population, as it measures all the three of these symptoms.

## Conclusions

As demonstrated by this targeted review of the literature in advanced-stage GC, there have been clinical studies in which significant or meaningful PRO differences were observed even in the absence of clinical differences. These findings suggest that PRO instruments may be sensitive to subtle treatment-related QOL or symptom benefits that cannot always be captured by common clinical instruments.

The EORTC QLQ-C30 along with the EORTC QLQ-STO22 module appears to provide the most comprehensive measurement of concepts important in GC with their extensive testing in GC patients, and this pair of instruments has the strongest body of psychometric evidence to support them. While the EORTC suite of instruments is the most widely used in advanced-stage GC, questionnaire length must be balanced against frequency of administration to minimise patient burden and missing data. Some of the brief PRO instruments developed for symptom assessment are administered as daily diaries, but this frequency of administration is likely too burdensome for GC patients. Patients with the poorest QOL and most severe symptoms would be the least likely to complete the instruments. Furthermore, the power afforded by the additional data to be modelled longitudinally as provided in daily diaries may be negated by the number of missing patient responses. For these reasons, the administration of PRO instruments at clinical study visits prior to treatment, rather than as daily diaries, is recommended. If the patient burden of the administration is of particular concern in a clinical study, the MDASI-GI could be considered in lieu of the EORTC QLQ-C30 and EORTC QLQ-STO22, as it includes fewer items and requires less administration time.

Future development of PRO instruments in advanced-stage GC should include topics of concern to patients without being overly burdensome due to length. Given the preliminary evidence that GC tumour anatomical location and, consequently, its symptomatology may be subjected to geographical variation, the analysis of efficacy and PRO data should include subgroup analysis by tumour location and geographic location in multicountry studies. In addition, given the prevalence of GC in Asian countries, there remains a concern that PRO instruments used in Asian studies have been linguistically translated, but not culturally adapted, from the instruments developed for use in Western countries and therefore do not measure all the elements of symptoms, symptom burden, and functional impacts in patients with GC.

GC symptoms and symptom burdens vary depending on a number of factors; however, this review supports the use of PROs, including evaluation of symptoms and symptom burdens, to show treatment benefit, even in the absence of clinical outcome differences. PRO instruments should be carefully selected for the study population and thoroughly translated, culturally adapted, and validated if being used in a language or culture other than the ones for which they were developed. Future development and validation efforts may be directed to shortening available scales to cover the concepts most important to patients with advanced-stage GC.

## Conflict(s) of Interest

Professor Evans has received honoraria, consultancy fees, and support to attend conferences from Bristol Myers Squibb. He has also received honoraria and consultancy fees from Bayer, Roche, Otsuka, and Clovis and he has received research funding from many pharmaceutical companies.

## Figures and Tables

**Table 1.** Overview of evaluated PRO instruments for advanced-stage GC: symptoms.

	EORTC QLQ C30 + EORTC QLQ-STO22	FACT-G + FACT-Ga	MDASI-GI
Symptoms of advanced GC			
Abdominal pain or discomfort	X	X	
Anorexia or appetite loss	X	X	X
Confusion or lack of concentration	X		X
Dysphagia or difficulty, pain, or inability to swallow	X	X	X
Vomiting	X		X
Weight loss	X	X	
Symptoms of GC (not identified by stage of disease)			
Bloating or abdominal swelling	X	X	
Bloating or early satiety or feeling full, bloated	X	X	X
Constipation	X		X
Diarrhoea	X	X	X
Dry mouth	X		X
Dyspepsia or indigestion, burping	X	X	
Dyspnea or respiratory issues, shortness of breath	X		X
Flatulence		X	
Fatigue, tiredness, weakness	X	X	X
Hair loss	X		
Heartburn	X		
Nausea	X	X	X
Pain (bodily)	X	X	X
Taste changes	X		X
Impacts of GC			
Anxiety, distress, feeling of impending doom	X	X	X
Body image, felt less attractive	X		
Daily activities or getting around	X	X	X
Depression, sadness	X	X	X
Difficulties eating or drinking	X	X	
Economic, financial	X		
Emotional functioning	X	X	X
Going to the toilet	X		
Sleep problems	X	X	X
Social activities, relationships	X	X	X
Trouble enjoying meals, trouble eating with others	X	X	
Worry about health or future, thinking about illness, lack of enjoyment	X	X	X
Work impairment	X	X	X

EORTC QLQ-C30: European Organisation for Research and Treatment of Cancer Quality of Life Questionnaire (core measure).

EORTC QLQ-STO22: European Organisation for Research and Treatment of Cancer Quality of Life Questionnaire-Gastric Cancer Module.

FACT-G: Functional Assessment of Cancer Therapy-General.

FACT-Ga: Functional Assessment of Cancer Therapy-Gastric Cancer.

MDASI-GI: MD Anderson Symptom Inventory-Gastrointestinal Cancer.

GC: gastric cancer.

PRO: patient-reported outcome.

**Table 2.** Overview of evaluated PRO instruments in advanced-stage GC: characteristics and psychometric evidence.

	EORTC QLQ-C30	EORTC QLQ-STO22	FACT-G	FACT-Ga	MDASI-GI
Concepts measured	Function (five subscales: physical, role, cognitive, emotional, and social) five symptoms (fatigue, nausea or vomiting, pain, dyspnea, diarrhoea), global health status and QOL, and financial impact	Symptoms: Dysphagia (four items) Pain or discomfort (three items) Upper GI symptoms (three items) Eating restrictions (five items) Emotional (three items) Dry mouth, hair loss, and body image	Physical well-being (seven items); social/ family well-being (seven items); emotional well-being (six items); functional well-being (seven items)	GC symptoms and impacts (e.g., pain, reflux, dysphagia, eating difficulties, tiredness, weakness, interference, and difficulty planning)	GI symptoms (constipation, diarrhoea, difficulty swallowing, change in taste, and feeling bloated), general cancer symptoms (13 items), and interference (six items)
Number of items/Time to complete/Response rate	30 (version 3.0) 10 min [24] Less than 4% missing for all items [34]	22 15 min for EORTC QLQ-C30 and QLQSTO22 [24] Less than 8% missing for all items [33]	27 (version 4) Time and response rate not reported	19 Time and response rate not reported	24 2–3 min Missing data rate of 0.18% [26]
Population of intended use	Cancer patients	Patients with GC, varying in disease stage, and treatment modality	Cancer patients	Patients with GC, varying in disease stage, and treatment modality	Patients with any GI cancer (not only GC)
Response options	28 items with a four-point Likert scale; two items with a seven-point numerical scale	Four-point Likert scale	Five-point Likert scale	Five-point Likert scale	11-point NRS
Recall period	Past week	Past week	Past seven days	Past seven days	Last 24 h
Scoring	Scores range from 0 to 100 (raw scores for items or summed subscales are transformed to 0–100) High score = worse symptoms or better function	Five subscale scores and three single-item scores High score = worse symptoms or more problems; further details not available	Subscale scores range from 0 to 24; from 0 to 28 according to the number of items in subscale Total score: 0–108 Higher score = better QOL	One subscale score range 0–76 Higher score = better QOL	Scores range from 0 to 10 for individual items Mean scale scores can be computed for the 13 symptom-severity items and for the six interference items
Internal consistency reliability	Yes [27]	Yes [24, 27, 33]	No evidence	Yes [25, 30]	Yes [26]
Test-retest reliability	No evidence	Yes [24, 34]	No evidence	Yes [25]	No evidence
Content validitya	Yes: cognitive debriefing [24]	Yes: concept elicitation [29] Yes: cognitive debriefing [24]	No evidence	Some patient inputs [30] Yes: cognitive debriefing [30]	Yes: patient interviews [26] Yes: cognitive debriefing [26]
Construct validity	Yes [27, 34]	Yes [24, 33, 34, 27]	No evidence	Yes [25]	Yes [26]
Ability to detect change	Some evidence [24]	Yes [24]	No evidence	Yes [25]	No evidence
Data-collection method	Self-administered; interviewer administered	No details available	Self-administered; interviewer-administered	No details available	No details available
Instrument adminis tration mode	Paper-pencil; computer-administered	No details available	Paper-pencil Computer-administered programs are under development	No details available	Paper-pencil
Cultural and language validation	Over 81 languages [35]	38 languages [35]	Over 53 languages [36]	Japanese translation [25, 30]	Chinese translation [37]

^a^Content validity as evidenced by patient involvement in concept elicitation and/or item generation.

EORTC QLQ-C30: European Organisation for Research and Treatment of Cancer Quality of Life Questionnaire (core measure).

EORTC QLQ-STO22: European Organisation for Research and Treatment of Cancer Quality of Life Questionnaire-Gastric Cancer Module.

FACT-G: Functional Assessment of Cancer Therapy-General.

FACT-Ga: Functional Assessment of Cancer Therapy-Gastric Cancer.

GC: gastric cancer.

GI: gastrointestinal.

MDASI-GI: MD Anderson Symptom Inventory-Gastrointestinal Cancer.

NRS: numerical rating scale.

QOL: quality of life.

PRO: patient-reported outcome.
